# High Prevalence and Low Diversity of *Rickettsia* in *Dermacentor reticulatus* Ticks, Central Europe

**DOI:** 10.3201/eid2804.211267

**Published:** 2022-04

**Authors:** Alena Balážová, Gábor Földvári, Branka Bilbija, Eva Nosková, Pavel Široký

**Affiliations:** University of Veterinary Sciences Brno, Brno, Czech Republic (A. Balážová, B. Bilbija, P. Široký);; Centre for Ecological Research, Budapest, Hungary (G. Földvári); Masaryk University, Brno (E. Nosková);; Central European Institute of Technology, Brno (P. Široký)

**Keywords:** Rickettsia, Rickettsia raoultii, *Dermacentor reticulatus* ticks, tick-borne infections, Dermacentor spp.-borne necrosis erythema lymphadenopathy, DEBONEL, tick-borne lymphadenopathy, TIBOLA, Czech Republic, Hungary, Slovakia

## Abstract

We collected 1,671 *Dermacentor reticulatus* ticks from 17 locations in the Czech Republic, Slovakia, and Hungary. We found 47.9% overall prevalence of *Rickettsia* species in ticks over all locations. Sequence analysis confirmed that all tested samples belonged to *R. raoultii*, the causative agent of tick-borne lymphadenopathy.

The ornate dog tick, *Dermacentor reticulatus*, is a proven vector of pathogens of public health and veterinary importance, including tick-borne encephalitis virus, Omsk hemorrhagic fever virus, rickettsiae, *Babesia* spp., and several others (*1*). *D. reticulatus* ticks are now expanding into new areas of northern and central Europe (*1*), where a higher prevalence of associated diseases can be expected.

Although intensively studied during the past decade, bacteria of the genus *Rickettsia* have been overshadowed by other tickborne pathogens of primary medical importance. Rickettsiae of the typhus group and spotted fever group (SFG) present the greatest health risks. The *D. reticulatus* tick is a vector for SFG rickettsiae. Among *Rickettsia* species, *R. raoultii* and *R.* *slovaca* are recognized as causative agents of rickettsioses with typical lymphadenopathies, called tick-borne lymphadenopathy or *Dermacentor*-borne necrosis erythema and lymphadenopathy (*2*), which are widespread in Eurasia (*1*). *R. helvetica*, which causes milder symptoms, was also reported from *D. reticulatus* ticks (*1*,*3*).

We analyzed 1,671 *D. reticulatus* ticks (851 female and 820 male) for prevalence, diversity, and distribution of SFG rickettsiae in the Czech Republic, Slovakia, and Hungary. Ticks were collected by flagging for previous studies conducted during 2009–2020 from 7 locations in the Czech Republic, 7 in Slovakia, and 5 in Hungary (Appendix). We selected places with a high abundance of *D. reticulatus* ticks for analyses*,* to promote high detection probability (Table). We used a duplex quantitative PCR method aiming for *gltA* gene fragments of *Rickettsia* (147 bp). We calculated prevalence (Sterne’s exact method if n <1,000, adjusted Wald method if n >1,000) and basic statistical comparisons in Quantitative Parasitology 3.0 (*4*). We also amplified fragments of 2 outer-membrane protein genes, *ompA* (590 bp) and *ompB* (475 bp), by conventional PCR and selected a subset of 5–10 positive samples from each location (144 total) for sequencing (Macrogen, https://www.macrogen.com) and identifying species (Appendix).

**Table:** Locations of *Dermacentor reticulatus* tick sampling and observed prevalence of *Rickettsia* spp., Central Europe

Location	Country	Year collected	Coordinates	No. positive/total no. collected	Prevalence% (95% CI)
Hodonín	Czech Republic	2020	48°51′22′′N 17°05′18′′E	64/90	71.1 (60.6–79.6)
Lanžhot	Czech Republic	2011	48°41′18′′N 16°59′22′′E	49/90	54.4 (43.9–64.5)
Lednice	Czech Republic	2009	48°49′08′′N 16°48′23′′E	20/90	22.2 (14.5–32.1)
Lednice	Czech Republic	2020	48°49′08′′N 16°48′23′′E	14/75	18.7 (11.2–29.2)
Mikulčice	Czech Republic	2009	48°47′57′′N 17°05′35′′E	66/90	73.3 (63.4–81.8)
Moravská Nová Ves	Czech Republic	2009	48°46′54′′N 17°04′36′′E	53/90	58.9 (48.3–69.0)
Moravská Nová Ves	Czech Republic	2020	48°46′23′′N 17°02′59′′E	44/90	48.9 (38.3–59.5)
Číčov	Slovakia	2011	47°46′28′′N 17°46′05′′E	40/90	44.4 (34.4–55.0)
Ďulov Dvor	Slovakia	2011	47°47′24′′N 18°10′14′′E	6/90	6.70 (3.0–13.8)
Jurský Chlm	Slovakia	2011	47°48′09′′N 18°31′01′′E	31/90	34.4 (24.9–45.0)
Klížska Nemá	Slovakia	2011	47°44′51′′N 17°49′42′′E	20/90	22.2 (14.5–32.1)
Klúčovec	Slovakia	2011	47°47′49′′N 17°43′29′′E	57/90	63.3 (52.8–72.9)
Lándor	Slovakia	2011	47°47′31′′N 18°08′03′′E	67/90	74.4 (64.5–82.7)
Studienka	Slovakia	2011	48°31′18′′N 17°08′02′′E	62/90	68.9 (58.4–77.9)
Dunaremete	Hungary	2011	47°53′33′′N 17°30′52′′E	39/90	43.3 (33.3–53.9)
Hévíz	Hungary	2013	46°47′14′′N 17°11′54′′E	53/90	58.9 (48.3–69.0)
Kisbodak	Hungary	2011	47°53′53′′N 17°30′31′′E	45/90	50.0 (39.4–60.6)
Kondorfa	Hungary	2006	46°53′42′′N 16°23′57′′E	29/77	37.7 (27.2–49.3)
Szendehely	Hungary	2017	47°50′60′′N 19°06′26′′E	41/79	51.9 (40.5–62.7)

We identified all isolates as *R. raoultii*. Our *ompA* gene sequences were 99.83% identical to haplotypes from Italy (GenBank accession no. HM161792.1) and Denmark (accession no. MF166732.1). We used *ompB* gene sequences to create a phylogenetic tree (Figure; Appendix) in which both sequences were placed into a highly supported subclade formed by sequences of *R. raoultii*. We did not detect either *R. slovaca* or *R. helvetica* at the locations in the study, but the prevalence of these species in *D. reticulatus* ticks is generally low because the main vectors are *D. marginatus* ticks for *R. slovaca* and *Ixodes ricinus* ticks for *R. helvetica* (*2*,*3*).

**Figure:**
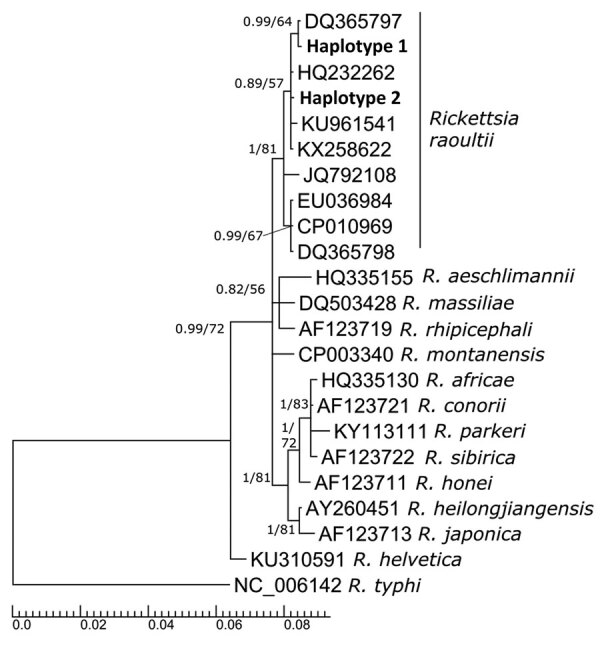
Phylogenetic tree inferred from outer membrane protein *ompB* region (600 bp) showing 4 separate branches of *Rickettsia* spp. in ticks. The sequences we obtained (bold) were placed into highly supported subclade corresponding with *R. raoultii*. First sequence (haplotype 1) shows 100% identity with GenBank accession no. DQ365797 from *D. reticulatus* ticks from France. Second sequence (haplotype 2) has 100% identity with GenBank accession no. HQ232262 from *D*. *reticulatus* ticks from Germany. The numbers at the nodes show posterior probabilities under the Bayesian inference/bootstrap values for maximum likelihood. GenBank accession number are provided for reference sequences. Branch lengths indicate expected numbers of substitutions per nucleotide site.

The mean prevalence of *Rickettsia* in *D. reticulatus* ticks was 47.9% (95% CI 45.5%–50.3%), without significant difference between sexes (p = 0.307 by χ^2^ test). Remarkably, we observed the lowest prevalence (6.7%) in Ďulov Dvor, Slovakia, ≈3 km from Lándor, which had the highest prevalence (74.4%) (Table). Differences in the surrounding environments might account for this discrepancy: Ďulov Dvor by an oxbow lake in the middle of arable land and Lándor in a forest along the river Váh. We assumed more abundant interconnected populations of host animals with unrestricted movement live in the forest environment. Data from Lednice, Czech Republic, situated in the middle of farmland, indicated ≈20% prevalence, consistently lower than the ≈60% in nearby areas of floodplain forests along the Morava River near Mikulčice. Comparing findings from the earlier and newer sample collections showed that the proportion of positive ticks remained consistent and variability over time was not significant. Specifically, we compared samples from Lednice (2009 and 2020; p = 0.574 by χ^2^ test), Moravská Nová Ves (2009 and 2020; p = 0.178 by χ^2^ test), and Mikulčice (2009) and Hodonín (2020), ≈9 km apart (p = 0.739 by χ^2^ test).

Distribution of the pathogen in *D. reticulatus* tick populations seems to be very uneven in Central Europe, which is also suggested by other studies (*5*). Our overall prevalence of 47.9% corresponds with similar data showing the prevalence of *R. raoultii* in *D. reticulatus* ticks to be 56.7% in Germany, 57.8% in Hungary, and 50.2% and 45.6% in 2 locations in Slovakia (*5*–*7*). On the other hand, researchers also found much lower prevalences of 10.8% in Slovakia (*8*) 15.6% in the Czech Republic (*3*) and 14.9% in Austria (*9*). Although significant seasonal differences in prevalence were reported (*10*), our data showed that the high observed prevalence in the study locations remained consistent over a long time period.

Our data suggest an overall high prevalence of *R. raoultii* and its possible long-term stability in *D. reticulatus* tick populations in the studied region, highlighting the enduring high risk of acquiring this rickettsial infection. Besides veterinary consequences (*1*), this risk should be considered by medical personnel and public health authorities because the incidence of tick-borne lymphadenopathy might increase with the reported (*1*) expansion of the vector into new areas and its growing abundance in Central Europe. 

Appendix Additional information about *Rickettsia* in *Dermacentor reticulatus* ticks in Central Europe
