# Supplementary material for: High Prevalence and Low Diversity of Rickettsia in Dermacentor reticulatus Ticks, Central Europe
Source: Emerg Infect Dis. 2022 Apr;28(4):893–5. doi: 10.3201/eid2804.211267 (PMC8962898; doi:10.3201/eid2804.211267)

Article DOI: https://doi.org/10.3201/eid2804.211267

High Prevalence and Low Diversity of *Rickettsia* in *Dermacentor reticulatus* Ticks, Central Europe

Appendix

Materials and methods

We isolated DNA from samples from the Czech Republic and Slovakia using alkaline hydrolysis in 1.25% NH_4OH (*1*) and samples from Hungary using a NucleoSpin tissue kit (Macherey-Nagel, http://www.mn-net.com) according to manufacturer instructions. For pathogen detection we ran real-time PCR using Probes Master mastermix on Light Cycler (both Roche Diagnostics, https://www.roche.com) and for sequencing we ran conventional PCR using PPP mastermix (Top-Bio, http://www.top-bio.com) and VWR Doppio gradient (VWR, https://www.vwr.com) (*2–4*) (Appendix Table).

Commercial provider Macrogen (https://www.macrogen.com) performed sequencing. We analyzed obtained sequences by BLAST algorithm (*5*) and aligned them using Geneious alignment with 21 relevant sequences downloaded from the GenBank database. We selected representative sequences of spotted fever group *Rickettsia* species based on BLAST analysis and phylogeny published elsewhere (*6*). We cut the final alignment to 600 bp and executed phylogenetic analysis in Geneious Prime software (*7*) with *Rickettsia typhi* (GenBank NC_006142) as an outgroup. We tested phylogenetic relationships by Bayesian inference analysis (*8*) and maximum likelihood analysis (*9*). We performed the Bayesian analysis by Geneious Prime plugin MrBayes version 3.2.6 (https://www.geneious.com) using the GTR (general time-reversible) substitution model for 10^6 generations, with trees and parameters sampled every 200 generations. We summarized the trees after removing 10% burn-in. We carried out the maximum likelihood analysis by the Geneious Prime plugin PhyML 3.3.20180621 using the GTR substitution model. We calculated nodal supports with 1000 bootstrap replicates and visualized the tree using TreeGraph 2.12.0 (*10*).

Appendix Table. Methods used for sample examination and preparation of gene fragments for sequencing*

Method	Target gene	Primers and probes, µmol/L	Sample volume/total volume, µL	Program				Ref
				Hotstart	Cycles	Final extension	Cooling	
Duplex quantitative PCR with probe	*gltA*	ApMSPf_upg 0.4<br>ApMSPr 0.4<br>gltA-CS-5 0.6<br>gltA-CS-6 0.6<br>5'Cy.5- ApMSPp-3'BHQ3 0.2<br>5'Hex- gltA-CS-3'BHQ2 0.4	2.0/20	95°C/10 min	45× [95°C for 10 s, 51°C for 30 s, 60°C for 30 s]	None	40°C for 10 min	(*2*)
PCR	*ompA*	Rr190.70p0.5<br>190.701 0.5	2.5/25	95°C/5 min	35× [95°C for 15 s, 54°C for 15 s, 72°C for 30 s]	72°C/3 min	10°C/ ∞	(*3*)
PCR	*ompB*	ompB.4362p 0.5<br>ompB.4836n 0.5	2.5/25	95°C/5 min	35× [95°C 15s, 54°C 15 s, 72°C 30 s]	72°C/3 min	10°C/ ∞	(*4*)

References

<jrn>1. Kubelová M, Tkadlec E, Bednář M, Roubalová E, Siroký P. West-to-east differences of *Babesia canis canis* prevalence in *Dermacentor reticulatus* ticks in Slovakia. Vet Parasitol. 2011;180:191–6. PubMed https://doi.org/10.1016/j.vetpar.2011.03.033</jrn>

<jrn>2. Balážová A, Baláž V, Ondruš J, Široký P. Duplex qPCR assay for detection and quantification of *Anaplasma phagocytophilum* and *Rickettsia* spp. Ticks Tick Borne Dis. 2020;11:101462. PubMed https://doi.org/10.1016/j.ttbdis.2020.101462</jrn>

<jrn>3. Regnery RL, Spruill CL, Plikaytis BD. Genotypic identification of rickettsiae and estimation of intraspecies sequence divergence for portions of two rickettsial genes. J Bacteriol. 1991;173:1576–89. PubMed https://doi.org/10.1128/jb.173.5.1576-1589.1991</jrn>

<jrn>4. Choi YJ, Lee SH, Park KH, Koh YS, Lee KH, Baik HS, et al. Evaluation of PCR-based assay for diagnosis of spotted fever group rickettsiosis in human serum samples. Clin Diagn Lab Immunol. 2005;12:759–63. PubMed</jrn>

<jrn>5. Altschul SF, Gish W, Miller W, Myers EW, Lipman DJ. Basic local alignment search tool. J Mol Biol. 1990;215:403–10. PubMed https://doi.org/10.1016/S0022-2836(05)80360-2</jrn>

<jrn>6. Sekeyová Z, Danchenko M, Filipčík P, Fournier PE. Rickettsial infections of the central nervous system. PLoS Negl Trop Dis. 2019;13:e0007469. PubMed https://doi.org/10.1371/journal.pntd.0007469</jrn>

<jrn>7. Kearse M, Moir R, Wilson A, Stones-Havas S, Cheung M, Sturrock S, et al. Geneious Basic: an integrated and extendable desktop software platform for the organization and analysis of sequence data. Bioinformatics. 2012;28:1647–9. PubMed https://doi.org/10.1093/bioinformatics/bts199</jrn>

<jrn>8. Ronquist F, Huelsenbeck JP. MrBayes 3: Bayesian phylogenetic inference under mixed models. Bioinformatics. 2003;19:1572–4. PubMed https://doi.org/10.1093/bioinformatics/btg180</jrn>

<jrn>9. Guindon S, Gascuel O. A simple, fast, and accurate algorithm to estimate large phylogenies by maximum likelihood. Syst Biol. 2003;52:696–704. PubMed https://doi.org/10.1080/10635150390235520</jrn>

<jrn>10. Stöver BC, Müller KF. TreeGraph 2: combining and visualizing evidence from different phylogenetic analyses. BMC Bioinformatics. 2010;11:7. PubMed https://doi.org/10.1186/1471-2105-11-7</jrn>

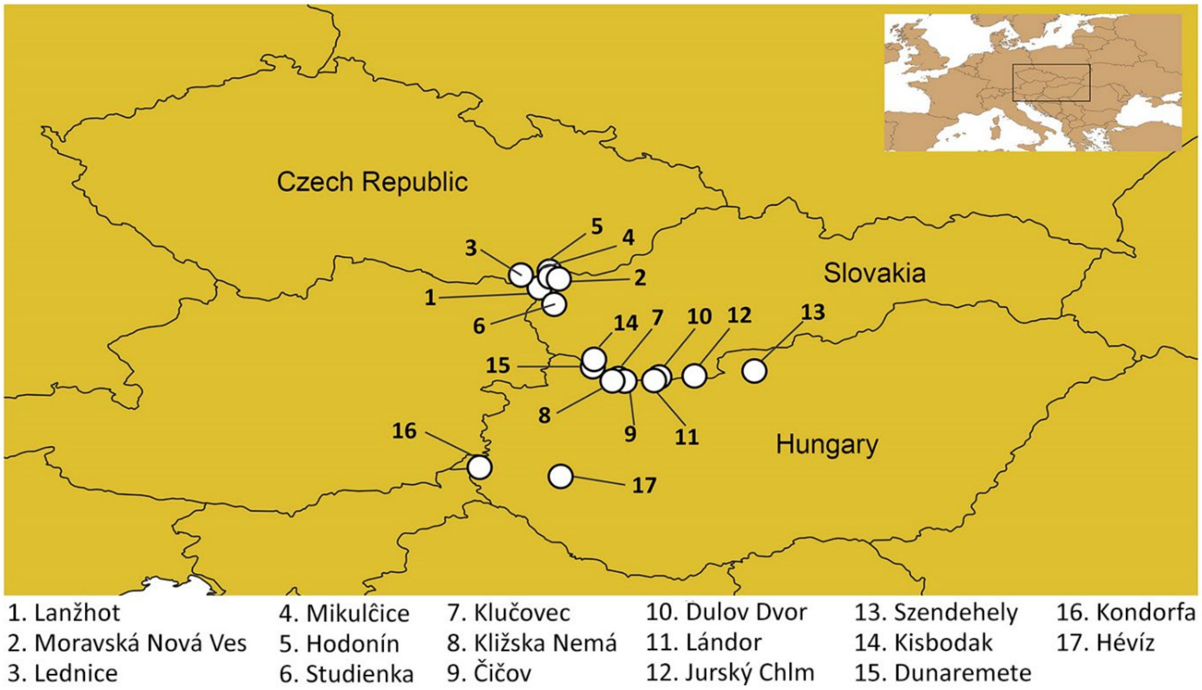

Supplement: Appendix — Additional information about Rickettsia in Dermacentor reticulatus ticks in Central Europe [file 21-1267-Techapp-s1.pdf]
